# Efficacy of omalizumab (Xolair®) in patients with moderate to severe predominately chronic oral steroid dependent asthma in Taiwan: a retrospective, population-based database cohort study

**DOI:** 10.1186/s12890-015-0156-2

**Published:** 2016-01-08

**Authors:** Hao-Cheng Chen, Chien-Da Huang, Erin Chang, Han-Pin Kuo

**Affiliations:** Department of Internal Medicine, Saint Paul’s Hospital, Taoyuan, Taiwan; Department of Thoracic Medicine, Chang Gung Memorial Hospital and Chang Gung University College of Medicine, Taipei, Taiwan; Master of Medicine Management (M.M.M.); Assistant HEOR Manager, Novartis, Taiwan; Department of Thoracic Medicine and Medical Education, Chang Gung Memorial Hospital, 199 Tun Hwa N. Rd., Taipei, Taiwan

**Keywords:** Omalizumab, Asthma, Population-based database, Cohort study, Real-life setting

## Abstract

**Background:**

Omalizumab (Xolair®), a recombinant monoclonal anti-IgE antibody, has demonstrated efficacy in clinical trials conducted in patients with moderate to severe persistent allergic asthma. We aimed to investigate the efficacy, discontinuation and medical resource utilization of omalizumab in the real-life setting in Taiwan.

**Methods:**

This study was a retrospective, population-based database cohort study using the Taiwan NHIRD from 2007 to 2011 assessing the efficacy of omalizumab therapy over 4 months on changes in asthma medication, asthma control, frequency of exacerbations and hospitalization rates at baseline and after omalizumab discontinuation.

**Results:**

There was a reduction in asthma medication post omalizumab therapy and severe exacerbations and hospitalizations from baseline (31.2 %, *n* = 282) to the end of follow-up (11.8 %, *n* = 144, *p* < 0.001). Nearly all the patients received chronic oral corticosteroids at baseline (92.4 %). The number of ER visits decreased from 1.13 ± 2.04 to 0.29 ± 0.83, and the mean number of admissions decreased from 5.93 ± 16.16 to 2.75 ± 12.02 from baseline to the end of follow-up (*p* < 0.001). After discontinuation of omalizumab, the cost of ER medical expenses decreased from New Taiwan dollars (NTD) 3934 at 2 months to NTD 2860 at 12 months.

**Conclusions:**

Patients who received omalizumab therapy for over 4 months were more likely to reduce the use of other asthma medications and less likely to experience an asthma exacerbation, ER visits, and hospitalization, even after the discontinuation of omalizumab. These data suggest that omalizumab has efficacy in improving health outcomes in patients with moderate to severe predominately chronic oral steroid dependent asthma in the real-life setting in Taiwan.

## Background

Asthma is a chronic inflammatory disease of the airways, and the symptoms can usually be relieved with inhaled corticosteroids (ICS) [1]. The majority of patients with asthma suffer from mild-to-moderate disease which may be relatively well controlled with the use of standard therapy. However, in 5 ~ 10 % of asthma patients the symptoms may continue to progress even with high-dose inhaled and oral corticosteroid treatment [2]. Patients with difficult-to-treat asthma may require repeated hospital admissions thereby incurring increased healthcare costs and affecting their working performance [3].

Evidence indicates that 50 % to 80 % of difficult-to-treat patients have an allergic component, with IgE playing a key role in triggering and maintaining allergic airway inflammation [4–8]. Omalizumab (Xolair®), a recombinant monoclonal anti-IgE antibody, has demonstrated efficacy in clinical trials conducted in patients with moderate to severe and severe persistent allergic (IgE-mediated) asthma in reducing the risk of exacerbations, hospitalization, and emergency room (ER) visits [4, 8]. Omalizumab was licensed in Taiwan in 2008 for patients aged 6 years and older with severe persistent allergic asthma that was inadequately controlled despite the use of high-dose ICS plus long-acting β2 agonists (LABA). Numerous randomized clinical trials have shown that adding omalizumab to current asthma therapy is effective and well tolerated [9–12]. Data from these clinical studies have shown that add-on therapy with omalizumab significantly reduces asthma exacerbations, use of ICS and ER/hospital visits. The results from a large cohort of patients with severe uncontrolled asthma showed that add-on omalizumab was associated with a significantly decreased risk of hospitalization or ER visits in patients with uncontrolled severe asthma in real-life practice [13]. Recently, a 2-year, international, post-marketing observation registry (eXpeRience) [14] was conducted to evaluate the real-world effectiveness, safety and use of omalizumab therapy in 943 patients with uncontrolled persistent allergic asthma. The results confirmed the effectiveness of omalizumab in improving asthma control, the number and severity of exacerbations, symptoms, lung function and healthcare utilization, both in a real-world setting and in a clinically controlled study setting.

The objective of this retrospective cohort study was to compare the effectiveness and benefits of long-term (>4 months) omalizumab treatment and those after discontinuation of treatment in real-life medical practice. Reductions in asthma exacerbations and utilization of asthma-related medical resources in Taiwanese patients with uncontrolled persistent allergic asthma were assessed using a population-based claims database.

## Methods

### Study design

This was a retrospective, database cohort study using the Taiwan National Health Insurance Research Database (NHIRD) from 2007 to 2011.

### Data sources

The NHIRD contains comprehensive claims records of outpatient and inpatient care of enrollees in the National Health Insurance (NHI) program in Taiwan, which covers over 99 % of the population in Taiwan (more than 23 million people). This health claims database includes health care data and medical utilization details as well as basic information from all hospitals contracted with the NHI program, and is updated annually. Each individual is assigned a unique identity number (hence longitudinal data are available), and the available information includes basic demographics, details of medical services, detailed information on prescribed medications, diagnoses from specialists’ referrals and hospital admissions.

The NHI Bureau has established a uniform system to control the quality of medical services and coding. The identification data of the beneficiaries in the NHIRD is scrambled to protect their privacy before they are released for research purposes. NHIRD was not freely available and Chang Gung Memorial Hospital (CGMH) and Novartis, Taiwan granted access to it. This study was supported by CGMH and Novartis, Taiwan [Grant XMRPG 3C1071] and was exempted from full review by the Institutional Review Board of CGMH (102-3778B).

### Sample size justification

Since this is a retrospective, descriptive study in which no formal statistical testing was performed, the use of point estimates and 95 % confidence intervals (CIs) served to quantify the level of precision. There were 46, 130, 156, 196 patients prescribed with omalizumab, respectively, based on which year they received it in the NHIRD claims database, allowing to quantify the patterns of omalizumab usage and other anti-asthmatic regimen in routine practice with sufficient precision (frequency, proportions and 95 % CIs were calculated).

### Patient population

Patients with allergic asthma (International Classification of Diseases, 9th Revision, Clinical Modification [ICD-9-CM] codes 493.0, 493.9, 493.1 and 493.8) were identified from the dataset. The source population consisted of all subjects who had at least one prescription for omalizumab as part of an anti-asthma regimen between January 1, 2008 and December 31, 2011. The index date was defined as the time of first prescription of omalizumab. Data were collected from 1 year before the initiation of omalizumab treatment to 4 years after treatment initiation or until the end of 2011, whichever came first. The patients who discontinued omalizumab remained in the database and were followed up until the end of the observation period. We were only evaluating patients who receive omalizumab > 4 months. We aimed to investigate the efficacy, discontinuation and medical resource utilization of omalizumab in the real-life setting in Taiwan. In addition, we evaluated the reduction in asthma medication post omalizumab therapy and severe exacerbations and hospitalizations from baseline to the end of follow-up longitudinally. Thus, we did not enroll a matched cohort not receiving omalizumab who are equally severe.

### Variables (outcomes of interests)

Demographics and patient characteristics recorded from the database included age at cohort entry, gender, asthma disease history, and diagnosis. Past medical history, previous asthma medication, co-morbidities, diagnosing/prescribing doctor’s specialty and level of institution were also recorded.

#### Omalizumab use and treatment observation

Data on the treatment regimen including dose, duration, prescription refill schedule/interval, change in dose and discontinuation were recorded. Information on the co-use of other asthma medication, previous medication, co-medications, and their doses, and whether or not they were used with omalizumab (ICS, LABA + ICS, oral corticosteroids [OCS], short-acting muscarinic antagonist [SAMA] and long-acting muscarinic antagonist [LAMA]) was also recorded. Data were collected at initiation, and then 4, 6, 8, and 12 months before discontinuing omalizumab, and 2, 6, and 12 months after discontinuing omalizumab. Discontinuation of therapy was defined as a gap in therapy of 56 days.

#### Treatment effectiveness

The number of asthma events, and asthma-related medical resource utilization including hospitalization, ER visits and medication use to manage acute exacerbations were recorded. The time to recurrence (exacerbation event) or worsening of asthma symptoms after discontinuing omalizumab treatment was also recorded. Other data including the number of clinically significant asthma exacerbations and severity of these exacerbations, and the use of OCS and other asthma maintenance medications and the reasons for discontinuing or changing omalizumab treatment defined as asthma exacerbations were also recorded. Severe exacerbation was defined as: ER visit or hospitalization >1 day with OCS >20 mg/day; Non-severe exacerbation was defined as: OCS >20 mg/day without ER visit or hospitalization.

### Data analysis

All the analysis was conducted using SAS statistical software, version 9.3 (SAS Institute Inc, Cary, NC). Exacerbations in baseline and follow-up period were expressed as percentage and compared by using Fisher’s exact test. Patient number and dosages, ER visit and hospitalization of using other asthma medication post omalizumab therapy were defined as continuous variables and were compared by using Fisher’s exact test. Mean changes from baseline for ER visits and hospitalizations post omalizumab therapy was expressed by frequency and using paired-*T* test for comparison. Data were summarized with respect to demographic and baseline characteristics, effectiveness, and reasons for discontinuing omalizumab treatment. Descriptive statistics were presented as number, mean, and standard deviation (SD) for continuous variables, and frequency, percentage, and 95 % CI for categorical variables.

In addition to the overall analysis, the evaluations were stratified according to treatment persistence (duration). The prescriptions, usage, co-medications, asthma-related events, number of acute exacerbations, medical resource utilization, and cost were analyzed and compared between different treatment persistence groups.

## Results

### Characteristics of the study subjects

Table 1 shows the characteristics of the study subjects. In total, 46, 130, 156, and 196 patients received omalizumab in 2008, 2009, 2010, and 2011, respectively, based on which year they received it in the NHIRD claims database,

**Table 1:** Characteristics of the study subjects

	2008	2009	2010	2011
Asthma				
New asthma patients	791,730	434,854	376,048	385,942
All asthma patients	791,730	813,806	828,300	899,375
MOWH asthma patients	785,831	808,008	822,800	878,220
SAA				
New SAA patients	46,982	30,828	29,632	28,195
All SAA patients	46,982	48,116	51,072	52,439
Omalizumab				
New omalizumab patients	46	90	69	77
All omalizumab patients	46	130	156	196

*MOHW* Ministry of Health and Welfare, *SAA* severe allergic asthma

### The prescribing pattern and duration of omalizumab treatment

**Fig. 1:**
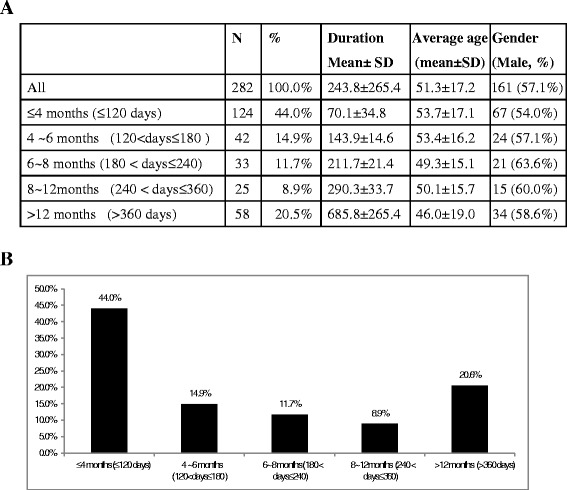
The duration and prescribing pattern of omalizumab: **a** The duration of omalizumab treatment: A total of 282 patients with moderate to severe asthma receiving omalizumab were enrolled. The mean duration of omalizumab treatment was 243.8 ± 265.4 days. **b** The prescribing pattern: Overall, 44 % of the patients received omalizumab therapy for less than 4 months with a mean duration of 70.1 ± 34.8 days, and 56 % of the patients received omalizumab for more that 4 months, including 15 % (4–6 months), 12 % (6–8 months), 9 % (8–12 months), and 21 % (over 12 months)

In total, 282 patients (161 male, 57.1 %) who received omalizumab had moderate to severe asthma with mean age of 51.3 ± 17.2 years. Nearly all the patients received chronic oral corticosteroids at baseline (92.4 %). The mean duration of omalizumab treatment was 243.8 ± 265.4 days, and 44 % of the patients received omalizumab for less than 4 months with mean duration of 70.1 ± 34.8 days (Fig. 1a). Of the remaining 56 % of the patients who received omalizumab for more than 4 months, 15 % received treatment for 4–6 months, 12 % for 6–8 months, 9 % for 8–12 months, and 21 % for more than 12 months (Fig. 1b).

### Decreases in other asthma medications post omalizumab therapy

At the end of follow-up, there was a significant decrease in the use of ICS, LABA/ICS, OCS, and SAMA (*p* < 0.001) as well as LAMA post omalizumab (*p* < 0.05) (Table 2). There was a reduction in all asthma medications compared to baseline 2 months before the discontinuation of omalizumab. The doses of LABA/ICS, OCS, and SAMA also decreased post omalizumab therapy (*p* < 0.001) (Table 3).

**Table 2:** Changes in other asthma medications post omalizumab therapy

Duration >120 days	Baseline^a^	Follow-up^b^	*p*-value^c^
*N* = 158	*N* (%)	*N* (%)	
ICS			<0.001*
With ICS	158 (100.0 %)	138 (87.3 %)	
Without ICS	0 (0.0 %)	20 (12.7 %)	
ICS + LABA			<0.001*
With ICS + LABA	157 (99.4 %)	124 (78.5 %)	
Without ICS + LABA	1 (0.6 %)	34 (21.5 %)	
OCS			<0.001*
With OCS	146 (92.4 %)	91 (57.6 %)	
Without OCS	12 (7.6 %)	67 (42.4 %)	
SAMA			<0.001*
With SAMA	49 (31.0 %)	4 (2.5 %)	
Without SAMA	109 (69.0 %)	154 (97.5 %)	
LAMA			0.027*
With LAMA	27 (17.1 %)	13 (8.2 %)	
Without LAMA	131 (82.9 %)	145 (91.8 %)	

^a^Baseline: 1 year before the index date

^b^Follow-up: 2 months before discontinuation (discontinuation of therapy was defined as a gap in therapy of 56 days)

^c^Fisher’s exact test, *P < 0.05

**Table 3:** Changes in LABA/ICS dosages, OCS, SAMA, and LAMA post omalizumab therapy

Duration >120 days	Baseline^a^	Follow-up^b^	Change from baseline	*p*-value^c^
*N* = 158	Mean ± SD	Mean ± SD	Mean ± SD	
ICS plus LABA				
Dose of salmeterol and fluticasone (mcg/day)	302.73 ± 236.28	215.82 ± 243.06	−86.91 ± 198.03	<0.001*
Dose of formoterol and budesonide (mcq/day)	162.72 ± 157.55	102.95 ± 149.38	−59.76 ± 138.84	<0.001*
OCS(tab/day)	1.99 ± 1.27	1.17 ± 1.66	−0.81 ± 1.61	<0.001*
SAMA(bottle/month)	0.44 ± 0.88	0.03 ± 0.21	−0.41 ± 0.86	<0.001*
LAMA(bottle/month)	0.15 ± 0.36	0.13 ± 0.47	−0.01 ± 0.47	0.6935

^a^Baseline: 1 year before the index date

^b^Follow-up: 2 months before discontinuation (discontinuation of therapy was defined as a gap in therapy of 56 days)

^c^Fisher’s exact test, *P < 0.05

### Decreased number of severe exacerbations post omalizumab therapy

There was a reduction in severe exacerbations or hospitalizations from baseline: 1 year before index day (31.2 %, *n* = 282) to follow-up: 2 months before discontinuation (Discontinuation of therapy was defined as a gap in therapy of 56 days.) (11.8 %, *n* = 144, *p* < 0.001). The number of ER visits and hospitalizations post omalizumab therapy decreased from 43.7 to 17.1 % and 34.8 to 17.7 %, respectively (*p* < 0.001) (Table 4). The mean number of ER visits decreased from 1.13 ± 2.04 to 0.29 ± 0.83 and the mean number of admissions decreased from 5.93 ± 16.16 to 2.75 ± 12.02 over the study period from baseline (1 year before the index date) to 2 months before the discontinuation of omalizumab (*p* < 0.001) (Table 5).

**Table 4:** ER visits and hospitalizations post omalizumab therapy

Duration >120 days	Baseline ^a^	Follow-up ^b^	*p*-value^c^
*N* = 158	*N* (%)	*N* (%)	
ER visit			<0.001*
Yes	69 (43.7 %)	27 (17.1 %)	
No	89 (56.3 %)	131 (82.9 %)	
Inpatient visit			<0.001*
Yes	55 (34.8 %)	28 (17.7 %)	
No	103 (65.2 %)	130 (82.3 %)	

^a^Baseline: 1 year before the index date

^b^Follow-up: 2 months before discontinuation (discontinuation of therapy was defined as a gap in therapy of 56 days)

^c^Fisher’s exact test, *P < 0.05

**Table 5:** Mean changes from baseline for ER visits and hospitalizations post omalizumab therapy

Duration >120 days	Baseline^a^	Follow-up^b^	Change from baseline	*p*-value^c^
*N* = 158	Mean ± SD	Mean ± SD	Mean ± SD	
ER visit				
(person × times/year)	1.13 ± 2.04	0.29 ± 0.83	−0.83 ± 2.05	<0.001*
Inpatient visit				
(person × times/year)	5.93 ± 16.16	2.75 ± 12.02	−3.18 ± 13.03	<0.001*

^a^Baseline: 1 year before index day

^b^Follow-up: 2 months before discontinuation (discontinuation of therapy was defined as a gap in therapy of 56 days)

^c^Mean change from baseline by paired *T* test, *p < 0.05

### Reductions in the dose of ICS + LABA, LABA and OCS, exacerbations, ER visits and cost after the discontinuation of omalizumab

**Fig. 2:**
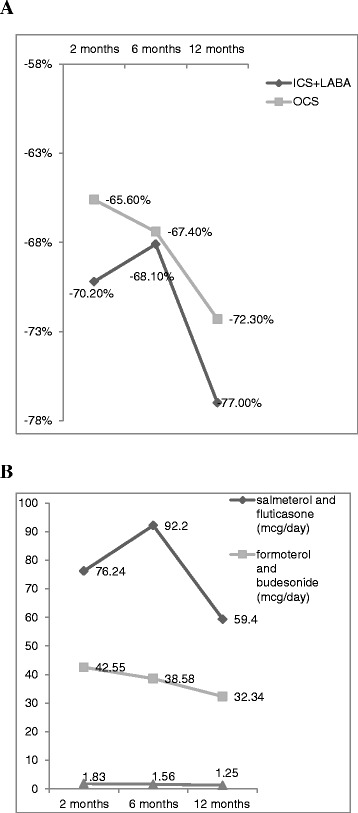
**a** Follow-up changes of ICS + LABA and OCS after the discontinuation of omalizumab (% change from baseline). Follow-up of changes in medication after the discontinuation of omalizumab for 12 months. There was a 68 % ~77 % reduction in ICS + LABA and a 65 % ~ 72 % reduction in OCS compared with baseline. Follow-up period: time point ± 14 days after omalizumab discontinuation; Time points: 2 months, 6 months, 12 months; Discontinuation of therapy was defined as a gap in therapy of over 56 days. **b** Dose of ICS + LABA and OCS after omalizumab discontinuation. The dose of ICS + LABA and OCS decreased after the discontinuation of omalizumab. Discontinuation of therapy was defined as a gap in therapy of over 56 days

**Fig. 3:**
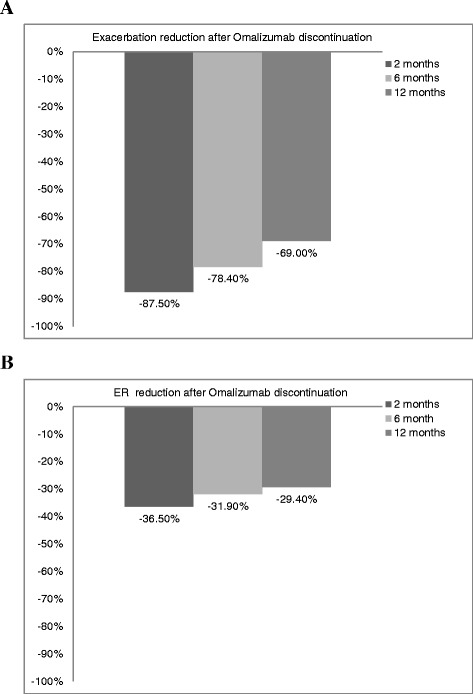
Changes in exacerbations and ER visits for the patients who experienced these events at baseline. **a** Exacerbation reduction: There was a 69.0 % ~87.5 % reduction in exacerbations after omalizumab discontinuation compared with baseline. **b** ER reduction: There was a 29.4 % ~ 36.5 % reduction of ER visit after omalizumab discontinuation compared with baseline

With regards to changes in medication after the discontinuation of omalizumab for 12 months, there was a 68 % ~77 % reduction in ICS + LABA, LABA and a 65 % ~ 72 % reduction in OCS compared with baseline (Fig. 2a). The doses of ICS + LABA, LABA and OCS after the discontinuation of omalizumab also decreased (Fig. 2b). There was a 69.0 % ~87.5 % reduction in the number of exacerbations (Fig. 3a) and a 29.4 % ~ 36.5 % reduction in ER visits (Fig. 3b) after the discontinuation of omalizumab compared with baseline. After the discontinuation of omalizumab, the mean cost of ER medical expenses decreased fro New Taiwan Dollar (NTD) 3934 at 2 months to NTD 2860 at 12 months.

## Discussion

This study was a retrospective, population-based database cohort study using the Taiwan NHIRD from 2007 to 2011. The patients who received omalizumab therapy for over 4 months were more likely to have reductions in other asthma medications and less likely to experience an asthma exacerbation-related ER visit and hospitalization. There were also reductions in the number of asthma medications, exacerbations and ER visits after the discontinuation of omalizumab at 2, 6, and 12 months compared with baseline. These results suggest that omalizumab is effective in improving health outcomes in patients with moderate to severe asthma in routine clinical practice in Taiwan. To the best of our knowledge, this is the first cohort study on this issue to use a national population-based database.

Omalizumab has been shown to be effective in double-blind placebo-controlled trials [15]. The reduction in the use of OCS observed in this study is in agreement with observations in a previous study [16]. Omalizumab may therefore decrease the potential for steroid-associated morbidities in patients with inadequately controlled severe allergic asthma. Molimard et al. suggested that omalizumab given to patients treated in a real-life setting provided a similar benefit to that observed in clinical trials [17], suggesting that the efficacy demonstrated in randomized controlled trials (RCTs) [8, 18–23] can be transposed to a real-life setting. A more recent study from Ireland [24] showed that the significant reduction in the number of exacerbations, hospitalizations and weekly need for rescue treatment validated the clinical benefits of omalizumab therapy in their population. The reduction in ICS and OCS use reflects improved symptom control, thereby facilitating stepping down from maximum standard therapy. A recent cohort study compared clinical outcomes 52 weeks pre- and post-omalizumab therapy, and the results showed that omalizumab treatment resulted in improved asthma control, with a significant reduction in asthma exacerbations and systemic steroid courses required and improvements in asthma control test (ACT) score [25]. The magnitude of the improvement of omalizumab in real-life setting was comparable to that observed in RCTs.

A retrospective analysis representing a preliminary report from the northeast of Italy reported that a moderate but statistically significant improvement in forced-expiratory volume in 1 s (FEV1), and an increasing proportion of exacerbation-free patients were observed after the initiation of treatment [26]. These findings were independent of the baseline severity of bronchial obstruction. A positive impact of omalizumab on rhinitis in patients with both asthma and rhinitis was also detected. Observed reductions in asthma-related events in particularly poorly controlled patients in an Italian [27] real-life setting are consistent with the results of other observational studies in France [17] and Germany [28]. In Israel, Rottem showed that omalizumab as an add-on therapy reduced the use of corticosteroids and improved the control of asthma, as evidenced by a reduced number of asthma-related ER visits [29]. Our study is compatible with the findings from these studies in Western countries, and it is the first study in Asia to use a nationwide database.

The mean duration of omalizumab treatment was 243.8 days in our study, however the optimal duration of omalizumab immunotherapy for responders who have benefited remains undetermined. There are limited data on asthma control after the cessation of omalizumab therapy. In the current study, 21 % of the patients received omalizumab therapy for over 12 months with a mean duration of 685.8 days, and persistent benefits were found after the discontinuation of omalizumab. In our results, there were reductions in ICS + LABA and OCS use in the patients who received over 4 months of omalizumab therapy for 12 months compared with baseline. There were also reductions in exacerbations and ER visits after the discontinuation of omalizumab for 12 months compared with baseline. Nopp et al. reported that there was no rebound phenomenon in the patients in whom omalizumab therapy was stopped after being treated for 6 years, and the patients reported that their asthma control continued to improve or remained unchanged when compared with being on treatment. Interestingly, the observed considerable down-regulation of basophil allergen sensitivity, cluster of differentiation (CD)-sens, which most likely represents mast cell allergen sensitivity, has been reported to contribute to clinical results [10]. Molimard et al*.* [30] published the results of a retrospective observational study on severe asthmatic patients after discontinuation of omalizumab therapy. Twenty-four lung specialists reviewed data from 61 responding patients who had discontinued omalizumab after a mean duration of 22.7 months of treatment. A loss of asthma control was documented in 34 patients (55.7 %) with a median interval between discontinuation and loss of control of 13.0 months. The discontinuation of omalizumab was not associated with any rebound effect or exacerbation of the disease, and control was sustained throughout the follow-up period of at least 6 months in nearly half of all patients, including all of those who had been treated for 3.5 years or more. After the reintroduction of omalizumab, 4 out of 20 patients did not respond again. The INNOVATE study (INvestigation of Omalizumab in seVere Asthma TrEatment) revealed that omalizumab withdrawal after 28 weeks of therapy led to the re-emergence of asthma symptoms, which correlated well with increasing free IgE and decreasing concentrations of the drug in serum. Reducing the dose of omalizumab below that in the dosing table was not recommended, as the resulting increase in free IgE would cause deterioration in asthma control [31]. However, a more recent study indicated that the withdrawal of omalizumab therapy after successful long-term therapy may cause severe asthma exacerbations [32]. In this study, for patients with at least 4 months of omalizumab therapy, there were reductions in asthma medications, exacerbations and ER visits after the discontinuation of omalizumab at 2, 6, and 12 months compared with baseline. A longer follow-up period may be warranted in future studies. The decision regarding cessation of omalizumab treatment should be undertaken individually after carefully weighing up the benefits and risks, especially in patients with a long history of severe asthma, and in those treated with high doses of OCS before omalizumab treatment is initiated. The high percentage of patients on oral steroids seems higher than other omalizumab for asthma studies, indicating they are more severe and complicated. Nearly all the patients received chronic oral corticosteroids at baseline (92.4 %). These data suggest that omalizumab has efficacy in improving health outcomes in patients with moderate to severe predominately chronic oral steroid dependent asthma in the real-life setting in Taiwan.

There are limitations in this retrospective, population-based database cohort study. A limitation of this study is that there is a major bias introduced by selecting successful patients who continued omalizumab for 4 months or more after initiation, especially when the drug could only be continued after 3 months in successful people. There is significant selection bias by excluding patients who did not complete at least 4 months of omalizumab. In total, 282 patients who received omalizumab had moderate to severe asthma. The mean duration of omalizumab treatment was 243.8 days, and 44 % of the patients received omalizumab for less than 4 months with mean duration of 70.1 days. We excluded the patients who did not complete 4 months of omalizumab therapy because some of the patients who discontinued omalizumab therapy had different confounding factors even though they were responders. However, because 40 % of patients who were taking omalizumab for less than 4 months, they may not have experienced a response if they would have taken it for greater than 4 months. It was hard to distinguish these patients because this was a retrospective, population-based database cohort study. In addition, as it costs much, NHI Bureau strictly audited the duration of omalizumab use every 3 months under controlled budget in Taiwan. 44 % of patients received omalizumab less than 4 months and 79 % less than one year in this study. Those who respond ascertain the value of continuous omalizumab use, whereas non-responders are likely to have other confounding factors such as poor adherence, persistent allergen exposure or other obstructive airway diseases [33]. Finally, we did not enroll a matched cohort not receiving omalizumab who are equally severe. The reasons for not doing this include that we aimed to investigate the efficacy, discontinuation and medical resource utilization of omalizumab in the real-life setting in Taiwan and evaluated the reduction in asthma medication post omalizumab therapy and severe exacerbations and hospitalizations from baseline to the end of follow-up longitudinally. The impacts and limitations should also be acknowledged including that the improvements in clinical outcomes reported may be due to other factors than treatment with omalizumab including regular asthma clinic review and improved adherence with asthma therapies.

## Conclusions

In summary, this study highlights patients who receive omalizumab therapy for over 4 months are less likely to experience an asthma exacerbation and hospitalization. Even after the discontinuation of omalizumab, the reduction of asthma medication, exacerbation and hospitalization were also observed. They were also more likely to require reduced maintenance oral and ICS therapy as well as the need for rescue therapy. Nearly all the patients received chronic oral corticosteroids at baseline (92.4 %). These population-based data suggest that omalizumab may be effective in improving health outcomes for patients with moderate and severe predominately chronic oral steroid dependent asthma in the real-life setting in Taiwan.

## Abbreviations

ACT: asthma control test
CD: cluster of differentiation
CGMH: Chang Gung Memorial Hospital
CI: confidence interval
ER: Emergency room
ICD-9-CM: International Classification of Diseases, 9th Revision, Clinical Modification
ICS: Inhaled corticosteroids
LABA: long-acting β2 agonists
LAMA: Long-acting muscarinic antagonist
NHI: National Health Insurance
NHIRD: National Health Insurance Research Database
NTD: New Taiwan Dollar
OCS: oral corticosteroid
RCT: randomized controlled trial
SAMA: short-acting muscarinic antagonist
SD: standard deviation
